# Personality affects defensive behaviour of *Porcellio scaber* (Isopoda, Oniscidea)

**DOI:** 10.3897/zookeys.515.9429

**Published:** 2015-07-30

**Authors:** Ivan Hadrián Tuf, Lucie Drábková, Jan Šipoš

**Affiliations:** 1 Department of Ecology and Environmental Sciences, Faculty of Science, Palacky University, Slechtitelu 27, CZ-77900 Olomouc, Czech Republic; 2 Department of Biology and Ecology, Faculty of Science, University of Ostrava, Chitussiho 10, CZ-71000 Slezska Ostrava, Czech Republic

**Keywords:** Anti-predatory behaviour, death feigning, thanatosis, predation, behavioural trait

## Abstract

We evaluated individual behavioural patterns of isopods expressed as tonic immobility following some intrusive treatments. Common rough woodlice, *Porcellio scaber*, were kept individually in plastic boxes and tested for tonic immobility repeatedly. Reactivity, sensitivity (number of stimuli needed to respond), and endurance of tonic immobility (TI) according three types of treatments (touch, squeeze, drop) were evaluated. Touch was the weakest treatment and it was necessary to repeat it a number of times to obtain a response; while squeeze and drop induced TI more frequently. Nevertheless, duration of the response persisted for a longer time with the touch treatment. Within each set of the three treatment, the strongest response was the third one, regardless of treatment type. Duration of reaction was affected by the size of the woodlouse, the smallest individuals feigning death for the shortest time. Despite body size, we found a significant individual pattern of endurance of TI among tested woodlice, which was stable across treatments as well as across time (5 repetitions during a 3 week period). *Porcellio scaber* is one of the first species of terrestrial isopods with documented personality traits.

## Introduction

Generally, when animals encounter their predator they (1) run away, (2) attack it or (3) stay invisible and/or look unpalatable.

Anti-predatory behaviour including boldness can be a part of animal personality. Personality of animals has been routinely studied during the last twenty years, although the study of personality in vertebrates prevails. Behavioural traits, which are consistent over time in individuals and as a response to different situations, have been described as a personality (Reale et al. 2007). The concept of personality has been used for a relatively broad spectrum of invertebrates including crustaceans (e.g. Briffa 2013, Biro et al. 2014, Brodin and Drotz 2014), but not explicitly studied in terrestrial isopods previously. The main behavioural traits found in Crustacea are boldness (Brifa et al. 2008; Hazlett and Bach 2010; Brifa and Twyman 2011; Brifa 2013), voraciousness (Biro et al. 2014) and activity (Yli-Renko et al. 2014).

Change in anti-predatory behaviour during growth and development of animal can challenge stability over time of the behavioural traits mentioned above. Examination of animal personality traits must consider consistency over two different time intervals: short intervals to determine whether behaviour is sufficiently consistent to be included in a study of personality, and longer intervals to determine how behaviour changes over the course of a lifetime (Stamps and Groothuis 2010).

During their evolution, terrestrial isopods colonised land and they were faced with new types of stresses, including new types of predators (Broly et al. 2013). The anti-predator mechanisms used by woodlice include escape, armour, cryptic colouration, chemical protection, acoustic warning, feigning death and/or specific posture (Witz 1990). Some of these strategies are not direct adaptations against predators, but evolved as parts of their terrestrial life-style. For example, escape is simply an extension of the ability to move as necessary to find food and mates, while armour is usually found in isopods living in (semi) dry conditions, which need to minimize water loss using thick cuticle (e.g. Smigel and Gibbs 2008; Csonka et al. 2013) and evolved as a defensive reaction. Chemical defensive secretions are a direct adaptation against predators being at least spider- (Gorvett 1956) and ant-repulsive (Deslippe et al. 1996; Yamaguchi and Hasegawa 1996).

Terrestrial isopods developed behavioural protection known as tonic immobility or death feigning, which is related also to behaviour known as “taking specific posture”. In general, the main difference between these categories is that “taking posture” is aimed for protection against being swallowed by a predator (e.g. Honma et al. 2006) and “feigning death” increases the probability to be ignored by predators with sight as the prevailing sense. This behaviour includes the so-called conglobation or volvation, behaviour typical for members of some isopod families such as Armadillidae, Armadillidiidae, or Cylisticidae, as well as for pill millipedes (Glomerida) and giant pill millipedes (Sphaerotheriida), some soil mites (Oribatida), and cuckoo wasps (Chrysidoidea). Conglobation involves the body being rolled into non-perfect or perfect ball with legs, antennae and ventral body surface more or less hidden. Non-perfect conglobation (e.g. typical for the genus *Cylisticus*) is less effective as uropods and antennae are not well protected. Nevertheless, tonic immobility is a much more general behaviour than conglobation and it is used by isopods (Quadros et al. 2012). Tonic immobility in non-conglobating forms of isopods is characterised by the contraction of the body and the contraction and folding of the legs towards the ventral side while holding the antennae folded or extended backwards and pressed against the dorsal part of the first pereonites (see fig. 1 in Quadros et al. 2012). During this posture the organism lacks motional responsiveness to external stimulation. Differences between death feigning and conglobation (called also shrinking in Anura) were discussed in the case of amphibians, but for isopods these differences are of marginal importance (Toledo et al. 2010).

The usefulness of feigning death as an anti-predatory behavioural strategy can theoretically be dependent on body size of an animal. If smaller animals can be easily overlooked by predator, the frequency of using this strategy by small animals can be higher than by bigger animals. This pattern was confirmed in some studies (Hals and Beal 1982; Quadros et al. 2012) but not found in other ones or in other species (Hazlett and Bach 2010; Quadros et al. 2012) for several crustaceans including terrestrial isopods.

We studied anti-predatory behaviour of the Common rough woodlouse *Porcellio scaber* Latreille, 1804, and we added a new parameter to standard experimental design – repetitions at the individual level. With this modification we were able to study the stability of behavioural traits, i.e. animal personality. The main aims of this research were: Are there any patterns in death feigning (tonic immobility, TI) behaviour? Is TI affected by type of treatment or its order? Is there a body-size pattern of behaviour among woodlice suggesting any developmental changes of its behaviour? Despite size of body, is there an individual specific pattern of behaviour among woodlice, i.e. are we able to evaluate their boldness on personal level?

## Methods

### Subjects and housing conditions

Several hundreds of Common rough woodlice, *Porcellio scaber*, were collected in the environment of Kutna Hora, Czech Republic (urban green areas and gardens) during June 2013. Following transport to laboratory, they were not sexed, but sorted in three size categories by length (small < 7 mm, medium 7–12 mm, and large > 12 mm). Size of woodlouse is related to its age (Zimmer 2002). Fifty individuals of each size category were inserted into small non-transparent plastic boxes (area 33 cm^2^) each with a thin plaster of Paris layer on the bottom. Each isopod individual had its own identifying code (ID) marked on its box. These codes enabled analyses of the stability of its behaviour (personality). Isopods were fed on potatoes and plaster was kept moist; natural (room) temperature regime was maintained at 21–26 °C.

### Procedure

Behavioural experiments followed the design used by Quadros et al. (2012); each isopod was exposed to several treatments. One experimental set contained three types of treatments to induce tonic immobility (TI): touch, squeeze, and drop. The touch stimulus was applied as gentle nudge to the isopod with forceps. The squeeze stimulus was applied as a firm grab to the isopod body by entomological soft-metal forceps, when one prong was undercutting the ventral part of the body and the other part was applied on the dorsal part. The drop stimulus was similar to squeeze one, though followed by lifting to *ca* 10 cm and then letting it drop back in the box.

**Figure 1.**
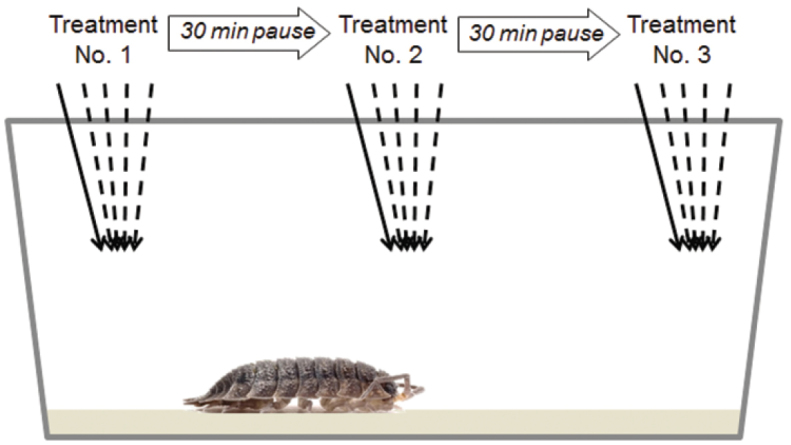
Design of one experimental set. Dashed arrows symbolise repeated stimuli applied if previous stimulus did not evoke tonic immobility. Experimental sets were applied repeatedly over a three week period; each individual was exposed to five experimental sets with 4 days intervals between.

The first treatment was applied and if TI was induced, its duration was measured. If necessary, the stimulus was repeated up to 5 times in order to induce TI. If TI was not induced, lack of reaction was recorded. We let individual woodlouse rest for approximately 30 minutes and applied the second treatment in the same way and the third treatment after a further half hour, respectively (Fig. 1). ID of woodlouse, order of types of treatments, sensitivity or promptness of TI induction (i.e. number of stimuli needed) or non-reactivity; and endurance of TI (i.e. time from start of TI to the first movement of antenna or leg) was measured in each experimental set. Each individual was involved in five experimental sets with 4 day intervals between experimental sets. The order of stimuli was changed systematically to distinguish the effect of type of stimulus from an effect of order of stimuli.

### Data analysis

We tested the effects of the three types of treatment (touch, squeeze and drop) on reactivity (presence/absence of reaction to stimulus, i.e. probability of inducing TI), sensitivity (number of stimuli needed to induce TI) and endurance of TI. Experimental sets which failed to induce TI were excluded from next data analyses. To determine the effect of different types of treatments we conducted repeated measures ANOVA. The error term of ANOVA reflects that we had the type of treatment nested within individuals of woodlice (ID). Data were not normally distributed therefore we transformed data by decimal logarithm. For multiple comparisons we used a pairwise t-test with adjusted p-values by the Holm correction. We used the F test to check the significance of the explanatory variables. Kendall’s coefficient of concordance was computed in order to determine the consistency of between-individual differences in the three types of treatment. We also used the correlation of TI endurance among different type of treatment. Significance of correlations was tested by using Kendall method with Bonferroni correction.

## Results

Three isopods died after the first experimental set, but data are available to evaluate from 738 experimental sets; TI as a reaction to at least one treatment was recorded in 334 sets (45% of sets) in 35 woodlice (23% of individuals). TI was induced by all treatments during the same experimental set in 41 experiments (6%) in 25 woodlice, with only one individual showing TI at each of the 15 treatments (i.e. through all five experimental sets).

**Figure 2.**
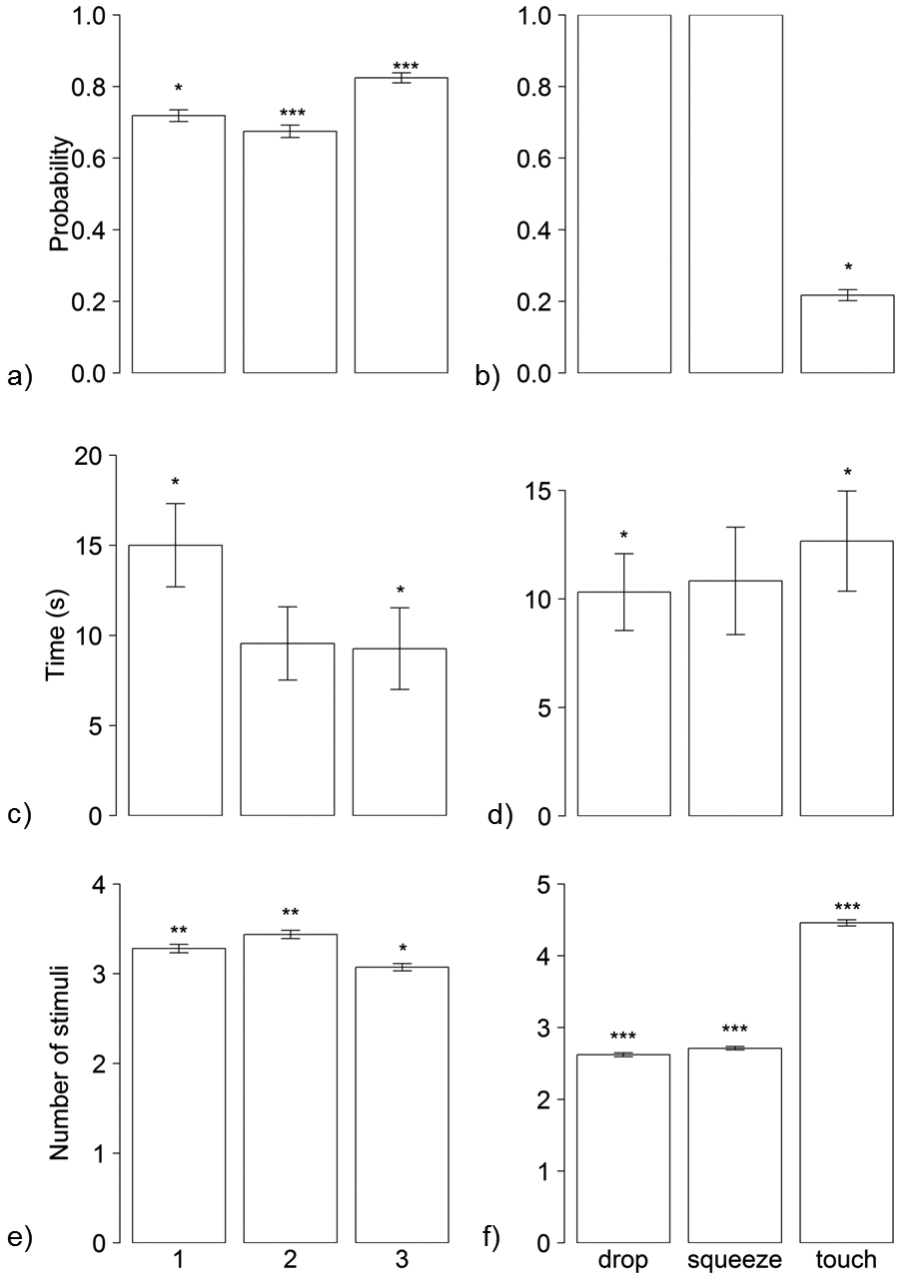
Tonic immobility of *Porcellio scaber* induced by different treatments: **a** probability of inducing TI by the first, the second and the third treatment **b** probability of inducing TI by different treatments **c** endurance of TI following the first, the second and the third treatment **d** endurance of TI following different treatments **e** sensitivity, i.e. promptness of inducing TI by the first, the second and the third treatment **f** sensitivity, i.e. promptness of inducing TI by different treatments. (*** p < 0.001; ** p < 0.01; * p ≤ 0.05)

If a woodlouse reacted to a treatment in an experimental set, the probability of reaction was influenced by type of treatment (F_2,298 _ = 1165.00, p < 0.001, Fig. 2b); in those experimental sets isopods reacted to drop and squeeze in all cases, but to touch in *ca* 20% only. If isopods reacted to treatment by TI, duration of TI significantly depended on the type of treatment (F_2,298_ = 2.97, p = 0.052, Fig. 2d), too: with touch followed by the longest TI. Also reactivity, i.e. number of stimuli needed to induce TI, was significantly dependent upon the type of treatment (F_2,298 _= 517.00, p < 0.001, Fig. 2f); if the global probability to react to touch is the lowest, more stimuli of touch were necessary to induce TI.

To avoid misunderstandings relating to the effect of treatment type and its order in the experimental set, the order of the applied treatments was changed. Without respect to type of treatment, the third treatment was the most probable to be followed by TI (F_2,298 _= 81.00, p < 0.001, Fig. 2a). Nevertheless the endurance of TI shortened significantly during experimental sets (F_2,298_ = 9.63, p < 0.001, Fig. 2c). On the other hand, number of stimuli needed to induce TI was significantly related to the order of the treatment (F_2,298 _= 16.55, p < 0.001, Fig. 2e).

**Figure 3.**
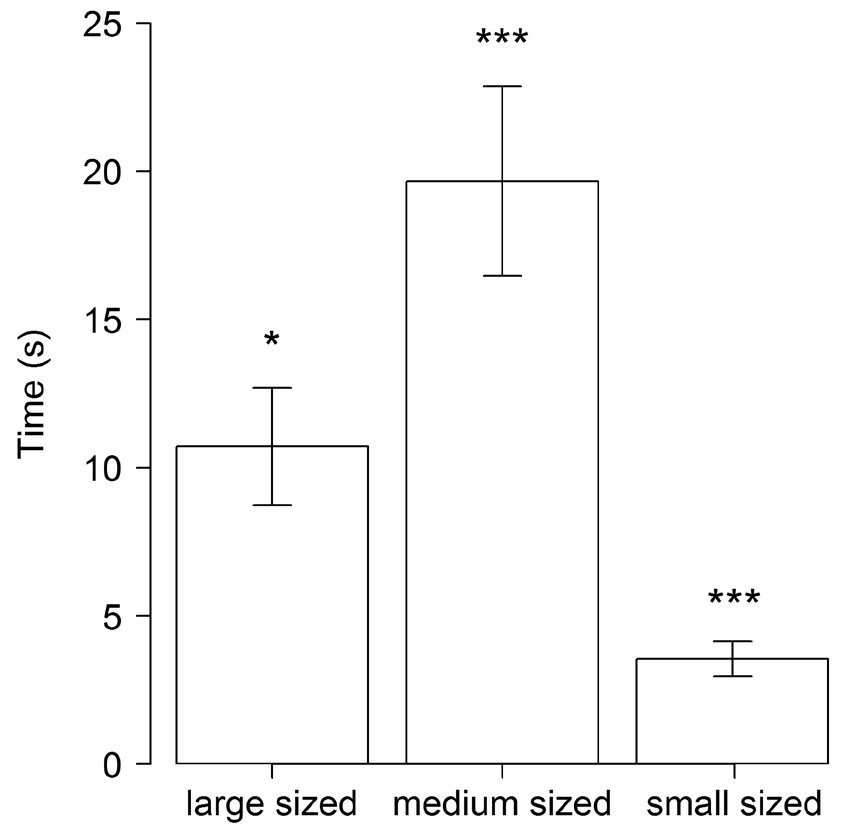
Endurance of tonic immobility of *Porcellio scaber* of different body sizes induced by treatments. (*** p < 0.001; ** p < 0.01; * p ≤ 0.05)

Although there were no significant differences among body-size categories of *Porcellio scaber* in the probability of inducing TI (F_1,148 _= 0.73, p = 0.395), the longest TI duration was performed by medium body sized woodlice (F_1,148 _= 6.75, p < 0.05, Fig. 3).

**Figure 4.**
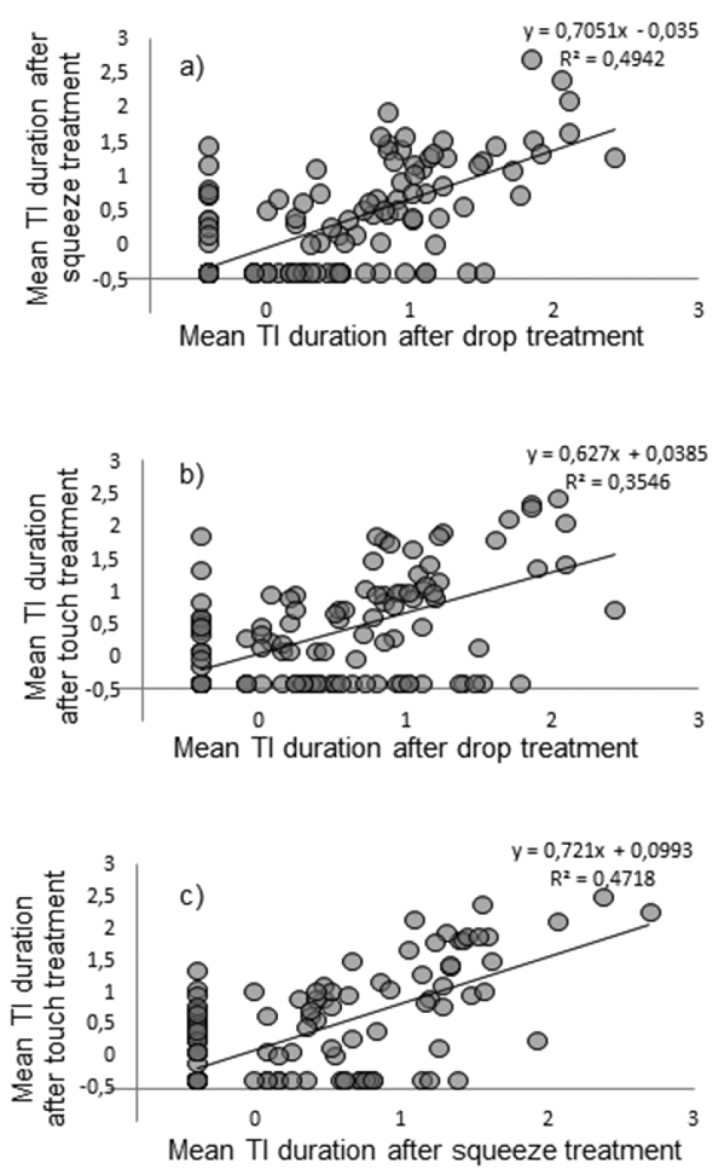
Correlations between duration (in seconds) of TI of *Porcellio scaber* induced by the different treatments: **a** correlation between duration of TI induced by squeeze and drop **b** correlation between duration of TI induced by touch and drop **c** correlation between duration of TI induced by touch and squeeze. Data were transformed by decimal logarithm.

Personality, i.e. individual stability of duration of TI was confirmed by Kendall’s concordance analysis for the whole reactive group of isopods (W = 0.73, p < 0.001); there were individual patterns of endurance of TI irrespective of type of treatment or its order. To avoid obfuscation of personality and size-dependent differences in behaviour, concordance analyses for individual size categories were calculated and revealed significant stability of endurance of TI inside all body-size categories (large size: W = 0.68, p < 0.001; medium size: W = 0.82, p < 0.001; small size: W = 0.65, p < 0.001). Stability of patterns of durations of TI can be visualised by correlations between endurances of different TI values (Fig. 4). Correlations between duration of TI were significant for *Porcellio scaber* analysed as a whole group as well as between different body-size groups (Table 1).

**Table 1.** Correlations between durations of TI of *Porcellio scaber* induced by different treatments: D – drop, S – squeeze, T – touch. (*** p < 0.001; ** p < 0.01; * p ≤ 0.05)

	all animals			large-size animals			medium-size animals			small-size animals		
	D	S	T	D	S	T	D	S	T	D	S	T
D	-	0.55***	0.45***	-	0.40*	0.32*	-	0.71**	0.56**	-	0.44*	0.44*
S		-	0.49***		-	0.61*		-	0.52**		-	0.32**
T			-			-			-			-

## Discussion

We evaluated reactivity, sensitivity, and duration of tonic immobility of *Porcellio scaber*. It is difficult to evaluate the functional significance of anti-predatory behaviour, as there are several interfering behaviours which affect probability of an animal being recognized, captured and consumed by predators (Lind and Cresswell 2005). These behaviours can have evolved independently; nevertheless it is impossible to measure the independent effect of one of those behaviours on the fitness of target animals. For this reason we cannot say that tonic immobility increases the fitness of *Porcellio scaber*.

Generally, the reactivity was relatively low (23% of isopods). It is known that tonic immobility is not the main anti-predatory strategy for *Porcellio scaber*, as a clinger ecomophological type (Schmalfuss 1984). They use sticking against a surface, or run away more frequently to escape predators (77% of woodlouse in our study tried to run away exclusively), or uses chemical protection (Gorvett 1956; Deslippe et al. 1996). Nevertheless, Quadros et al. (2012) found *Porcellio dilatatus* to be a highly responsive species (89% specimens used TI).

Reactivity of isopods was affected by the type of treatment. Whereas drop and squeeze were followed by TI regularly, touch was not an effective treatment for TI in some specimens. The explanation can be found in the manipulation of isopods by different kinds of predators (e.g. Sunderland and Sutton 1980, Dejean 1997, Řezáč and Pekár 2007, Quadros et al. 2012). Despite the lack of experimental verification of tonic immobility as defence behaviour against predators and regarding the size of *Porcellio scaber*, we can hypothesise that drop treatment is probably more similar to manipulation by some vertebrate visual predator (birds, amphibians, or lizards). Squeeze can be similar to manipulation by some small vertebrate or large invertebrate predators (e.g. small rodents or shrews, ground beetles of the genus *Carabus*), whereas touch resembles the manipulation of small invertebrate predators (spiders, centipedes, ants, etc.). According to these categories of predators, TI following drop can be very useful, if the isopod is lost by predator in leaf litter. Big predators do not loose time for looking for one small prey item. They probably continue walking and searching for another prey. Similarly, TI as response to squeeze can also help the attacked isopod to survive, if the predator is not able to manipulate the immobile prey very well. By contrast, the most effective defensive strategy against small invertebrate predators is the secretion of chemicals (Deslippe et al. 1996), which may not be so effective against larger predators.

It is necessary not to forget that *Porcellio scaber* is strongly thigmotactic (e.g. Friedlander 1964) and lives in large aggregations (Broly et al. 2012): this is important for two aspects concerning its TI reaction. First, touch is a common stimulus in the way of life of woodlice. In aggregates, there are many conspecific individuals around; reacting by TI to each touch becomes meaningless. For this reason, low reactivity and low sensitivity to touch is understandable. But if touch is repeated several times (it was necessary to repeat it more times than drop or squeeze), endurance of TI is longer than TI following drop or squeeze. This is probably because of the foraging mode of the predator: small invertebrate predators such as spiders or ants can manipulate small isopods for a longer time and can wait for the first movement (providing time to attack the un-armoured ventral side). Larger predators do not waste time by waiting; they swallow prey immediately if they notice and catch it.

Another advantage of aggregates is the higher probability of being passed over by a predator among running conspecifics (Miyatake et al. 2009). If larger predator turns over the shelter of a group of isopods (e.g. stone or bark on dead stump), it can be useful to stay in TI and wait until the predator is lured away by other, running members of the aggregation. It can be gainer strategy even if the woodlouse is lost by the predator (drop or squeeze). In addition, the shorter duration of TI can be more useful if the lured-away predator is coming back to search for the last prey items. It can be an explanation for higher reactivity and shorter endurance for TI following drop and squeeze stimuli.

Studies have shown changes in behaviour according to the type of disturbing treatment. Carbines et al. (1992) studied the character of escape mechanisms of isopods from predators. They measured turn alteration in a simple labyrinth and related it to the probability of survival (as direction of run). If the treatment was harmless cotton-wool fluff, the probability of survival was much lower than if *Dysdera* spider predators were the agent of disturbance. It means an authenticity of stimulus affected its defensive behaviour; perhaps over time in our prolonged experiment the authenticity of disturbance was decreasing. During one experimental set in our study, the reactivity increased in the third treatment while in the third treatment duration of TI was reduced. This resembles a situation when the isopod is (hypothetically) able to evaluate the meaningless stimulation of the experimenter and learn “to escape” from this situation by a more prompt TI response for a shorter time. As this “explanation” is rather implausible, shorter duration of TI in the last stimulus can be explained also by quick habituation of *Porcellio scaber* to stable environmental cues, as was described by Anselme (2013). Habituation, i.e. changes of response to repeated stimulus was reported also for the aquatic crab *Chasmagnathus granulatus* (Tomsic et al. 2009).

Although our research is not the first to look into TI in terrestrial isopods, the results presented here enable to test repeatability of responses of individual isopods, i.e. its personality. The concept of personality was used for behavioural studies of some Crustacean species, mainly Decapoda, i.e. in crabs, hermit crabs, crayfishes (e.g. Briffa 2013, Biro et al. 2014, Brodin and Drotz 2014), as well as Isopoda (Yli-Renko et al. 2014). Among terrestrial isopods, the only study dealing with personality known to the authors was done by Matsuno and Moriyama (2012). They found a correlation between the walking speed and endurance of conglobation in some specimens of the Common pill bug *Armadillidium vulgare*. Nevertheless, this “stable internal factor” was found only in specimens showing a stable-style end of conglobation: specimens that finished conglobation in two trials by leg movement or antenna movement consistently, were more “brave” (shorter duration of tonic immobility) and ran faster compared to leg-antenna “alternators” (i.e. specimens ending conglobation by antenna movement and leg movement in two trials). We also found correlations in individual specimens for duration of TI across different types of treatment and these correlations were found over three weeks (five experimental sets with 4 day breaks), meaning that there were some consistently more “bold” woodlice (short TI) and some more “shy” woodlice (long TI).

Correlations between length if TI, even if there is a decrease of endurance of TI during one experimental set, can be caused by habituation of isopods to repeated treatment as well as their sensitivity to new type of treatment. Anselme (2013) found that *Porcellio scaber* individuals are able to habituate to an environment in around 10 minutes. Over this time, they become less interested in stable stimulus and their activity decreased. In the same study woodlice preferred new stimuli (such as a new texture of substrate) if it was provided (Anselme 2013), or a random pattern of known stimuli (Anselme 2015), so there is some evidence of “curiosity” in *Porcellio scaber* (although not studied at individual level).

Documented “boldness”, as a parameter of personality of *Porcellio scaber*, is independent of size (age) of specimen. Hals and Beal (1982) reported that the largest specimens (> 1 cm of length) of *Porcellio scaber* reacted by TI at less intensity compared to smaller specimens (< 1 cm). Similarly Quadros et al. (2012) found the same pattern in reactivity for *Balloniscus sellowii*. However we did not find significant differences in reactivity of woodlice in our three body-size groups, although there are differences in endurance of TI among groups. The longest reaction time was measured in medium-sized woodlice (7–12 mm) and shortest in small-sized woodlice (< 7 mm). One explanation could be sought in terms of changes of the effectiveness of TI as a defence mechanism against predators. TI is not necessary for large woodlice against medium-sized and smaller predators, because large woodlice are less catchable and can use chemical defence: their glands are well developed and able to produce sufficient amount of secretions (Gorvett 1956, Sutton 1970) in comparison to the less developed glands in smaller stages of *Porcellio scaber*. As well TI would not be a successful protection for the smallest woodlice against predators such as *Carabus* or centipedes, as they are easy to manipulate. Indeed, the mortality of juvenile stages of isopods is estimated to reach 80% (Sutton 1970) and 11–51% decrease in populations is caused by predation upon juveniles by invertebrates (Sunderland and Sutton 1980). This indicates TI can be a useful strategy mainly for medium-sized *Porcellio scaber* specimens.

Besides finding differences in endurance of TI between body size groups, we also identified personal behavioural patterns in all tested individuals, as well as variation within these body-size groups. These findings are not able to resolve if personality is changing during individual development or not. Although behavioural traits can be stable across short time intervals, changes to personality due to development can cause inconsistency in responses to stimuli over longer time intervals (Stamp and Groothuis 2010). We did not evaluate if traits remained the same over long time intervals, but this type of stability was not proved for marine isopod *Idothea baltica* recently (Yli-Renko et al. 2015). Investigation of long-time stability of behavioural traits in terrestrial isopods should be a possible goal of future studies.
